# Case Report: Implantation of Dedifferentiated to Poorly Differentiated Thyroid Carcinoma After Endoscopic Thyroid Surgery

**DOI:** 10.3389/fonc.2022.896942

**Published:** 2022-05-06

**Authors:** Jin Hu, Xia Xu, Shuntao Wang, Fang Dong, Ximeng Zhang, Jie Ming, Tao Huang

**Affiliations:** ^1^ Department of Breast and Thyroid Surgery, Union Hospital, Tongji Medical College, Huazhong University of Science and Technology, Wuhan, China; ^2^ Department of Pathology, Union Hospital, Tongji Medical College, Huazhong University of Science and Technology, Wuhan, China

**Keywords:** thyroid carcinoma, endoscopic thyroidectomy, breast implantation, poorly differentiated thyroid carcinoma, case report

## Abstract

**Background:**

Endoscopic thyroidectomy is widely accepted for its advantages. However, implant metastasis remains a significant complication of endoscopic thyroidectomy.

**Methods:**

This is the first report of breast implantation diagnosed with poorly differentiated thyroid carcinoma following endoscopic thyroidectomy.

**Results:**

We present a case of a 35-year-old woman who was initially diagnosed with a 3.0 cm conventional papillary thyroid carcinoma after endoscopic thyroidectomy *via* total areola. Two years later, she was reported to have recurring poorly differentiated thyroid carcinoma in the right areola. Implantation after endoscopic thyroidectomy is rare, and even rarer is dedifferentiated papillary thyroid carcinoma around the implant site.

**Conclusions:**

Stringently evaluated endoscopic surgery indications, appropriate preoperative evaluation, meticulous surgical technique, and adequate protective measures can significantly reduce the incidence of local implantation or recurrence.

## Introduction

Minimally invasive thyroid surgery is the future of thyroid surgery. Patients widely accept endoscopic thyroidectomy (ETC) because it leads to less postoperative pain, faster postoperative recovery, and better cosmetic effect. Due to less trauma and fewer complications from ETC, surgeons have used it to treat benign and malignant thyroid disorders (1–3). However, ETC complications, such as local recurrence and implantation, remain significant issues. Several publications reported these complications (4–7). Here, we present a 35-year-old woman who suffered local implantation metastasis in her chest wall and was diagnosed with papillary thyroid carcinoma (PTC). Her right breast was diagnosed with poorly differentiated thyroid carcinoma (PDTC) after ETC.

While PDTC was a distinct clinicopathologic entity in the 1980s (8), it was not identified as a unique pathological entity by the World Health Organization until 2004 and by the Turin proposal until 2006 (9). PDTC is an aggressive thyroid cancer with an extremely low incidence and an intermediate survival rate.

## Case Report

**Figure 1:**
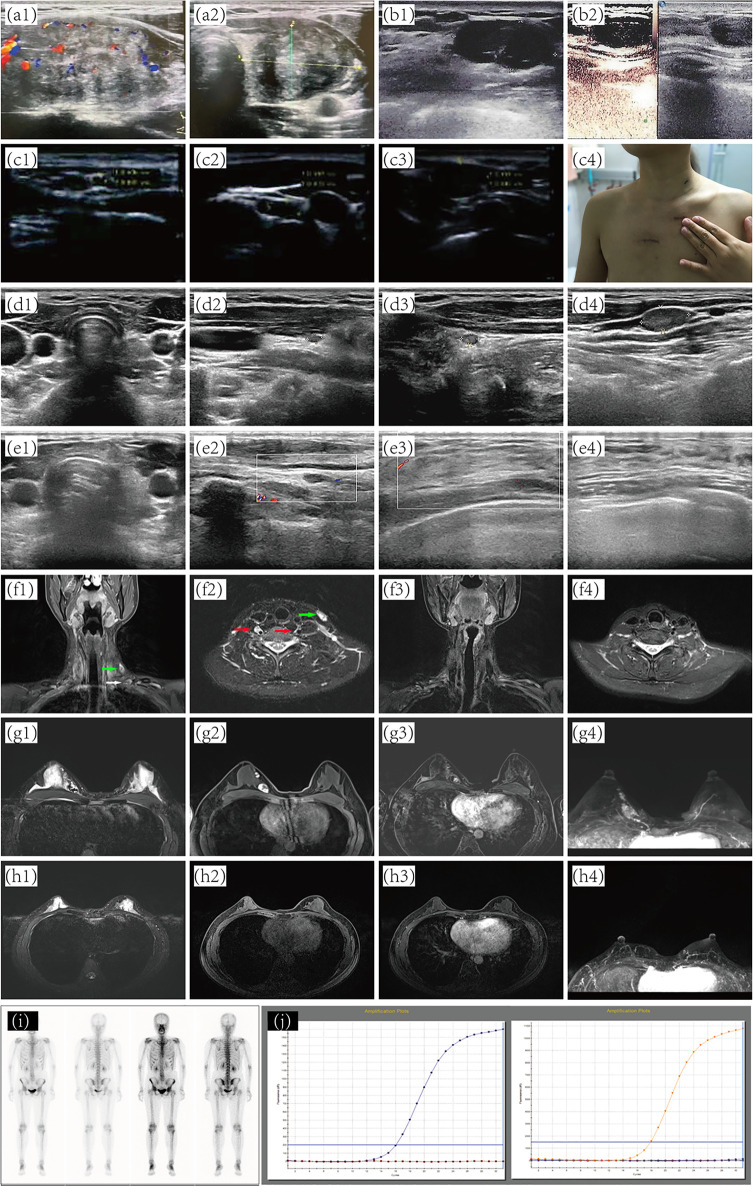
Preoperative and postoperative imaging diagnosis. **(A)** preoperative thyroid sonography. **(B)** Subcutaneous nodules around the right areola of the breast. **(C)** separate nodules in the right chest wall. **(D)** Ultrasound assessment of thyroid and cervical lymph nodes after patient receiving ETC. Ultrasound of thyroid **(E1, 2)** and breast **(E3, 4)** at five years post-operation. **(F1, 2)** metastatic lymph nodes. **(F3, 4)** No abnormality was found at five years post-operation. **(G)** A predominantly cystic mass with solid portions. No abnormality was found on MRI **(H)** and ^131^I-whole-body scan **(I)** at five years post-operation. **(J)** NRAS, BRAF, and PIK3CA genes by quantitative real-time PCR. ETC, Endoscopic thyroidectomy; MRI, magnetic resonance imaging.

In February 2014, thyroid sonography showed a tumor in the left thyroid region of a 35-year-old woman. The size was about 3.0 × 2.0 cm (**Figures 1A1, A2**), and the primary diagnosis was left thyroid adenoma. Therefore, the woman had her left thyroid nodule excised endoscopically, *via* total areola, at her local hospital. However, an intraoperative frozen section examination revealed that she had PTC. The patient then underwent open surgery with a total thyroidectomy and bilateral central neck dissection. Nevertheless, a postoperative pathological examination subsequently showed that she had left papillary carcinoma (**Figure 1D**) without any form of neck lymph node metastasis.

**Figure 2:**
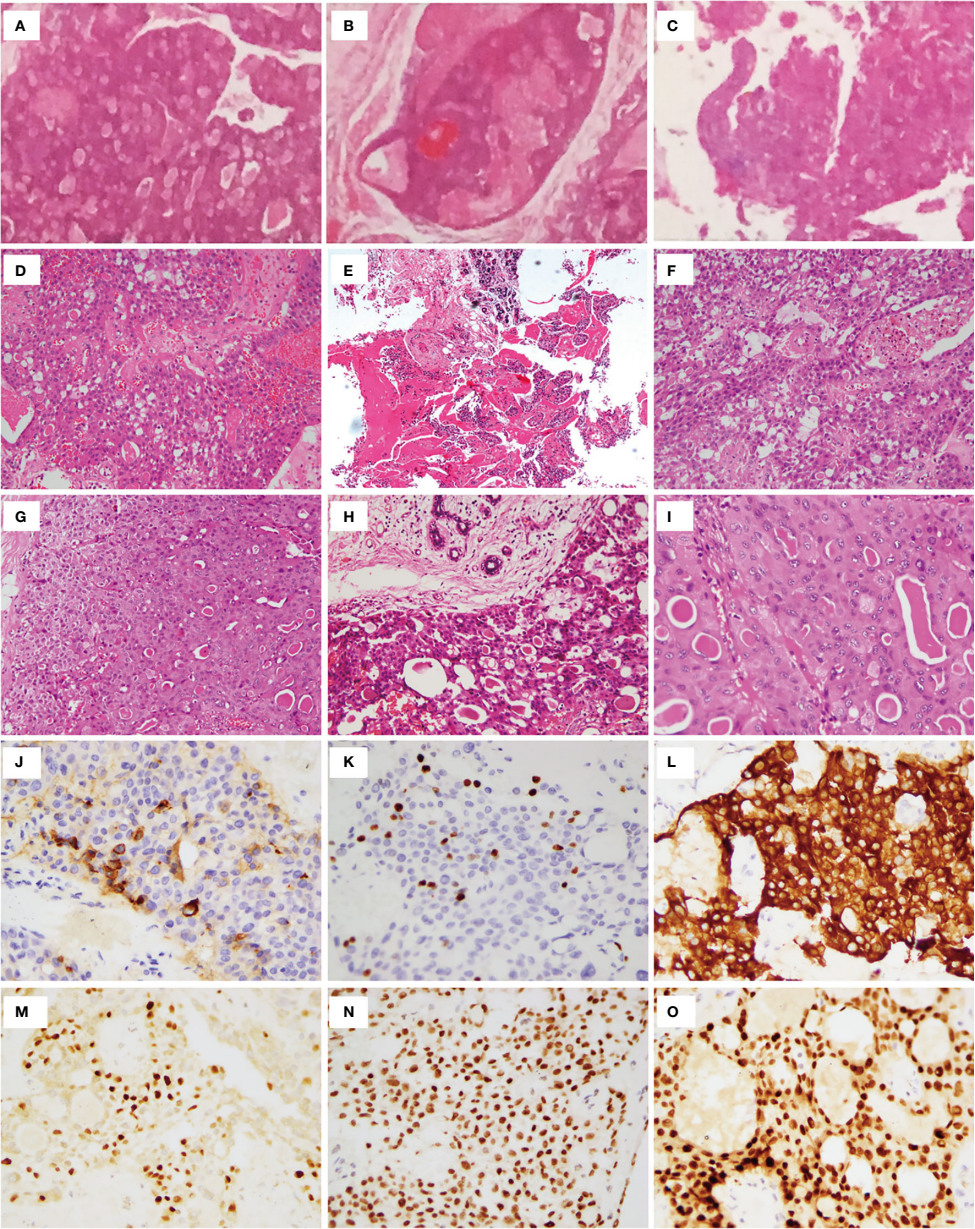
Histopathologic examination. Postoperative pathologic examination of nodules in the chest wall showed that all nodules contained PTC tissues **(A–C)**. **(D)** Total thyroidectomy (in 2014) revealed PTC without reporting cell features. **(E)** lesion of right breast. Left lateral cervical **(F)** and left supraclavicular fossa **(G)** lymph node excision revealed metastatic PTC. Nodule of right chest wall **(H)** and Subcutaneous tissue of left neck **(I)** excision revealed metastatic PTC. **(J)** TG. **(K)** Ki67. **(L)** CK-19. **(M)** GATA-3. **(N)** TTF-1. **(O)** Pax8.

In January 2016, PET/CT scanning indicated that the bilateral sternocleidomastoid, submandibular, and supraclavicular areas harbored multiple enlarged lymph nodes. Intense FDG uptake was also observed in multiple nodules located in the upper chest wall. Additionally, there were nodular and hyper-intense calcification shadows without FDG uptake in the upper inner quadrant of the left breast and the lower inner quadrant of the right breast of the patient. Sonography revealed four separate nodules in the right chest wall of sizes of 2.5 ×2.5 cm, 2.0 ×1.6 cm, 3.0 ×1.8 cm, and 2.5 × 2.0 cm, all located along the previously operated tunnels (**Figures 1C1–3**). Therefore, to remove those nodules in the right chest wall, the woman underwent local-tumor excision (**Figure 1C4**). A postoperative pathological examination showed that all nodules contained PTC tissues (**Figures 2A–C**), and immunohistochemical phenotyping showed positive expression of TTF-1, TG, galectin-3, and vimentin. The patient recovered well after the second operation and received postoperative adjunctive treatment.

Two months after the second operation, a ^131^I whole-body scan showed intense FDG uptake in the neck. The patient then came to our hospital in July 2016 with PTC complaints following surgery. Contrast-enhanced ultrasonography suggested that subcutaneous nodules around the right areola of the breast were metastatic tumors (**Figures 1B1, B2**). Sonography revealed lymph nodes metastatic in the neck (**Figure 2D**). A magnetic resonance imaging (MRI) examination revealed a metastatic tumor in the neck (**Figures 1F1, F2**) and the upper inner quadrant of the right breast (**Figures 1G1–4**). Enhanced computed tomography did not indicate metastatic nodules on the lungs. Preoperative coarse needle aspiration biopsy for the right breast nodule confirmed metastatic thyroid carcinoma. A third operation was, thus, performed on the patient. Modified radical neck dissection on the bilateral neck and partial mastectomy plus intraoperative frozen section were performed under general anesthesia. The final diagnosis of the right breast nodule was PDTC with breast metastasis by postoperative permanent pathological examination (**Figure 2E**). Left lateral cervical and left supraclavicular fossa lymph node excisions also revealed metastatic PTC (**Figures 2F, G**), and excisions of the nodule of the right chest wall and subcutaneous tissue of the left neck revealed metastatic PTC (**Figures 2H, I**). Immunohistochemical phenotyping showed positive TTF-1, TG, galectin-3, CK19, PCK, HBME-1, Pax8 expressions and negative ER, TPO, BRAF, calcitonin, Syn, CgA, and P63 expressions (**Figures 2J–O**). Combined with clinical characteristics and previous thyroid surgery history, the final diagnosis was PDTC with breast implantation. No mutations were detected in the NRAS, BRAF, and PIK3CA genes by quantitative real-time PCR (**Figure 1J**). According to the final reports (**Figures 1E1–4, F3, F4, H1–4, I**) issued in December 2020, the general conditions of the patient were favorable following that last operation. At the time, she was receiving TSH suppression treatment with levothyroxine.

## Discussion

The history of minimally invasive thyroid surgery shows that minimally invasive surgery on the neck was first attempted by Gagner et al. (10) in 1996. ETC then rapidly developed in the ensuing decades. Local recurrence and implantation after ETC were first described by Jung et al. (6) in 2008. However, a few rare local implantation cases have been reported; specifically, PTC dedifferentiated to PDTC presenting as implantation in the breast is rare.

Preoperative ultrasound examination suggested a huge thyroid nodule in the left thyroid region, but local surgeons still chose endoscopic surgery without distinguishing between benign and malignant nodules. The intraoperative frozen section examination revealed that she had PTC. The patient then had to wait to be received immediately after the open surgery. Unfortunately, two years after surgery, local implantation in the right chest wall and right breast was discovered. The pathological diagnosis of the latter was poorly differentiated carcinoma (a tumor size of 2.5 cm × 1.5 cm). The removal of tumor cell-contaminated instruments and ruptured mass(es) from the cavity and tunnel might result in tumor cell seeding in incision and chest tunnel.

Even though the mass is benign, it is necessary to achieve the safe removal of the tumor with an intact capsule. Caution should be taken during the operation to prevent the mass from being pinched or ruptured. To prevent the tumor cell spread, surgeons immediately removed the tumor(s) using a small retrieval pouch. During the endoscopic surgery, the mass was resected uneventfully. If we could not take it out of the incision, we should choose to expand the incision, or even switch to open surgery to remove it, followed by double-distilled water rinsing.

ETC should be done with great caution, especially for large or subcapsular tumors. Although percutaneous transthoracic needle biopsy of the mass is the specific method to obtain a definitive diagnosis before the operation, this does not influence the choice of surgery as a treatment. Unless the nodules have high-risk features such as an extra-thyroid extension or lymph node metastasis, the guidelines agree that fine-needle aspiration (FNA) should be performed on nodules at least 1 cm in size (11, 12).

If endoscopy is the initial diagnostic modality of choice, and FNA was discarded to obtain a definitive diagnosis, it seems unwise (namely, if the result of the specimen based on the intraoperative pathological frozen section is benign, the surgeon continued and completed their procedure. If it is malignant, surgeons choose to convert from endoscopic surgery to open surgery). This seemingly reduces the waste of medical resources and the suffering. However, once implantation occurs, the economic burden and psychological stress are greater for patients.

PDTC is a biologically very aggressive tumor with intermediate biological and clinical behavior between differentiated thyroid carcinoma and undifferentiated (anaplastic) thyroid carcinoma. It is defined by the World Health Organization (WHO) using criteria established in the Turin proposal. In the study by Thiagarajan et al. (13), the accuracy of FNAC-diagnosed PDTC was only 8.7%. The ultimate diagnosis relies on permanent pathological examination and immunohistochemical phenotyping. In patients with PTC, some biomarkers are positive, namely, CK, TG, and TTF-1; Syn and CgA are negative. Several other biomarkers, such as S-100, CK19, RET, and AR, are alternative biomarkers to diagnose PTC. TTF-1 and TG expressions in PDTC were positive, but TG expression was limited to island tumor cells and underdeveloped follicles. Immunohistochemical markers associated with the replication of thyroid cells can show proliferation. However, at present, in terms of immunohistochemical markers, the disease has no clear specificity. PDTC in young patients represents a genetically homogeneous group of tumors developing *via* molecular pathways not similar to adults. The latter commonly harbors initiating BRAF or RAS mutations (14). However, PDTC of this adult patient implanted in the breast did not detect NRAS, BRAF, or PIK3CA mutations.

Our report has several limitations. First, the patient underwent three operations in different hospitals, leading to the lack of some information, such as intraoperative images; second, in the period from the time when she underwent the first operation to now, some data may be lost, such as the results of continuous monitoring for TG. But this report also has an advantage. This is the first report of implantation lesions undergoing dedifferentiation after endoscopic thyroidectomy.

Our case presented several lessons that are worth sharing. Firstly, fine needle aspiration is a commendable procedure for preoperative evaluation, especially when the mass might be suspected of malignant nature by ultrasound examination. Secondly, the tumor was so large that the instrument could have broken it; in other words, the larger the tumor, the more possible the implantation. Surgeons should highly prioritize protecting tumors from breakage. Thirdly, if a tumor is broken, open surgery should be performed to obliterate it.

## Conclusion

Surgeons and patients widely accept ETC because of its advantages. Implantation after ETC is not typical. However, our case and currently reported cases worldwide, remind us that endoscopic surgery should be chosen cautiously for thyroid diseases. Open surgery, whenever required, must be handled stringently, even if only to take out the tumors.

## Data Availability Statement

The original contributions presented in the study are included in the article/**Supplementary Material**. Further inquiries can be directed to the corresponding authors.

## Ethics Statement

Ethical review and approval was not required for the study on human participants in accordance with the local legislation and institutional requirements. The patients/participants provided their written informed consent to participate in this study. Written informed consent was obtained from the individual(s) for the publication of any potentially identifiable images or data included in this article.

## Author Contributions

JH composed the manuscript. XX provided figures and pathology review. SW, FD, and XZ had the acquisition, analysis or interpretation of data for the work. JM and TH revised it critically for important intellectual content, final approval of the version to be published, and agreement to be accountable for all aspects of the work in ensuring that questions related to the accuracy or integrity of any part of the work are appropriately investigated and resolved. All authors listed have made a substantial, direct, and intellectual contribution to the work and approved it for publication.

## Conflict of Interest

The authors declare that the research was conducted in the absence of any commercial or financial relationships that could be construed as a potential conflict of interest.

## Publisher’s Note

All claims expressed in this article are solely those of the authors and do not necessarily represent those of their affiliated organizations, or those of the publisher, the editors and the reviewers. Any product that may be evaluated in this article, or claim that may be made by its manufacturer, is not guaranteed or endorsed by the publisher.
